# Supplementary material for: Physiological Responses during Parental Conflicts as Potential Biomarkers for Adolescent Depression
Source: Children (Basel). 2023 Jul 10;10(7):1195. doi: 10.3390/children10071195 (PMC10378688; doi:10.3390/children10071195)
Supplement: Supplementary file 1 [file children-10-01195-s001.zip › children-2460916-supplementary.pdf]

Anxiety and Depression Assessment Scales:

1. HAMD & HAMA

The HAMD scale is a commonly used clinician-rated tool for assessing the severity of depressive symptoms, which can effectively evaluate the severity of depression, with higher scores indicating more severe depression. The scale has good reliability and validity, with reliability coefficients ranging from 0.78 to 0.99, and a coefficient reflecting the authenticity of symptom severity of 0.92 [1]. The HAMA scale can be used clinically to assess anxiety symptoms, with the total score reflecting the severity of anxiety symptoms. The reliability coefficients for each item score range from 0.83 to 1.0, and the validity coefficient is 0.36. The Chinese version of the MINI assessment has good reliability and validity, with a test-retest reliability of 0.97-1.0, sensitivity of 91.2-100%, and specificity of 85.7-96.4% [2].

2. PHQ-9 & GAD-7.

The Chinese version of PHQ-9 has good reliability and validity in adolescents, with a Cronbach's α coefficient of 0.85, a test-retest reliability of 0.88 after 4 weeks, and a diagnostic cut-off score of 10 points, with a sensitivity of 93.33% and a specificity of 96.83% [3]. The Chinese version of GAD-7 has good reliability and validity in patients in a comprehensive hospital outpatient clinic, with a Cronbach's α coefficient of 0.898 and a test-retest reliability of 8.856. When the threshold value is set at 10 points, the sensitivity and specificity are 86.2% and 95.5%, respectively [4].

Mini International Neuropsychiatric Interview (MINI)

The MINI assessment is a structured interview tool used for screening and diagnosing Axis I psychiatric disorders in accordance with DSM-4 and ICD-10 criteria. The Chinese version of the MINI has demonstrated good reliability and validity. Test-retest reliability ranges from 0.97 to 1.0, while sensitivity ranges from 91.2% to 100% and specificity ranges from 85.7% to 96.4% [5].

Family Functioning Assessment Scales: FAD & SSFD

The Chinese version of FAD has good reliability and validity in the clinical measurement of family functioning, with an overall internal consistency coefficient of 0.91[6]. In this study, the overall family functioning was assessed using the total functional component scale, which includes 12 items as a tool for evaluating overall family functioning. SSFD is a tool developed in China to assess individual and family-level dynamic characteristics of family members. The revised version includes 23 items in four dimensions: family atmosphere, individualization, systemic logic, and disease concept. The scale has good reliability and validity, with Cronbach's α coefficient and split-half correlation coefficient of 0.79 and 0.84, respectively [7,8]. The scores in the four domains and the total score of the scale are positively correlated with the criterion-related measures.

Other Scales: CTQ、SES & CD-RISC

1. **CTQ**: The Childhood Trauma Questionnaire (CTQ) in its English version was developed by Professors Bernstein DP and Fink L from New York, USA. After being translated into

Chinese, it has demonstrated good reliability and validity when used in the Chinese adolescent and child population. The overall Cronbach's α coefficient of the Chinese version of the CTQ questionnaire is 0.77, and the Cronbach's α coefficients for each subscale range from 0.41 to 0.68. The test-retest reliability after two months is 0.75, and the test-retest reliability for each subscale ranges from 0.27 to 0.73 [9].

2. **SES**: Self-Esteem Scale (Chinese version). Both domestic and international studies have consistently reported high reliability and validity for the use of the SES measure. Among the 48 articles that employed the SES measure, reliability coefficients ranged from 0.59 to 0.9065, with all measures of internal consistency and split-half reliability exceeding 0.70. While there may be fewer studies reporting on the validity of SES measures in domestic research, the reported reliability coefficients have reached statistical significance, aligning with findings from international studies and indicating good reliability and validity of the SES measure[10].
3. **CD-RISC**: The Connor-Davidson Resilience Scale, translated by Xiao Nan and Zhang Jianxin, is designed to assess positive psychological qualities that promote individual adaptation to adversity. It consists of 25 items, rated on a 5-point scale ranging from "0" to "4" to indicate the degree of agreement with each statement, ranging from "never" to "almost always". The English version of the scale has demonstrated good internal consistency with a coefficient of 0.89, and a test-retest reliability coefficient of 0.87. Similarly, the Chinese version has shown strong internal consistency with a coefficient of 0.91, meeting the requirements of psychological assessment standards [11].

References

1. Hamilton, M. A rating scale for depression. *J. Neurol. Neurosurg. Psychiatry* **1960**, *23*, 56–62.
2. Hamilton, M. The assessment of anxiety states by rating. *Br. J. Med. Psychol.* **1959**, *32*, 50–55.
3. Hu, X.; Zhang, Y.; Liang, W. Reliability and validity of the patient health questionnaire-9 in Chinese adolescents. *Sichuan Ment. Health* **2014**, *27*, 357–360.
4. Qu, S.; Sheng, L. Diagnostic test of generalized anxiety scale in screening generalized anxiety disorder in outpatient department of psychology department of general hospital. *Chin. Ment. Health J.* **2015**, *29*, 939–944.
5. Si, T.; Shu, L.; Dang, W.; Su, Y.; Chen, J.; Dong, W.; Kong, Q.; Zhang, W. Clinical reliability and validity of the Chinese version of the Brief International Neuropsychiatric Interview. *Chin. Ment. Health J.* **2009**, *23*, 493–497.
6. Li, R.; Xu, F.; Ji, L.; Zhang, W. Preliminary Revision of the Family Functioning Rating Scale. *China J. Health Psychol.* **2013**, *21*, 996–1000.
7. Yang, J.; Kang, C.; Zhao, X. The Self-rating Inventory of Systematic Family Dynamics: Development, Reliability and Validity. *Chin. J. Clin. Psychol.* **2002**, *10*, 263–265.
8. Yu, L.; Zhao, X.; Zeng, W.; Tan, J.; Yu, Y.; Zeng, J.; Wan, C. Reliability and validity analysis of the revised version of the Systematic Family Dynamics Self-evaluation Scale in the test of Guangdong students. *Chin. J. Health Stat.* **2014**, *31*, 979–981.
9. Zhao, X.; Zhang, Y.; Li, L.; Zhou, Y.; Li, H.; Yang, S. Reliability and validity of the Chinese version of childhood trauma questionnaire. *Chin. J. Clin. Rehabil.* **2005**, *9*, 105–107.
10. Tian, L. Shortcoming and Merits of Chinese Version of Rosenberg (1965) Self-Esteem Scale. *Psychol. Explor.* **2006**, *26*, 88–91.
11. Gao, Z.; Yang, S.; Margraf, J.; Zhang, X. Wagnild-Young Reliability and Validity Test for Wagnild and Young's Resilience Scale(RS-11) in Chinese. *China J. Health Psychol.* **2013**, *21*, 1324–1326.
